# Cracking the riddle of dedifferentiated liposarcoma: is EV-MDM2 a key?

**DOI:** 10.18632/oncoscience.497

**Published:** 2020-02-01

**Authors:** Lucia Casadei, Raphael E Pollock

**Affiliations:** ^1^The James Comprehensive Cancer Center, The Ohio State University, Columbus, Ohio, USA; ^2^Department of Surgery, Division of Surgical Oncology, The Ohio State University Wexner Medical Center, Columbus, Ohio, USA

**Keywords:** dedifferentiated liposarcoma, extracellular vesicles, MDM2, MMP2, tumor microenvironment

## Abstract

Dedifferentiated liposarcoma (DDLPS) is molecularly characterized by wt p53 and MDM2 gene amplification causing MDM2 protein over-production, the key oncogenic process in DDLPS. Commonly located in fat-bearing retroperitoneal areas, almost 60% of DDLPS patients undergo multifocal recurrence, typically amenable to palliative treatment only, and occasionally develop distant metastasis. These factors lead to an abysmal 10% 10 year overall survival rate. Tumor cell-derived extracellular vesicles (EVs) can facilitate loco-regional malignancy dissemination by depositing molecular factors that participate in the development of pre-metastatic niches for tumor cell implantation and growth. High number of MDM2 DNA molecules was identified within EVs from DDLPS patient serum (ROC vs normal; 0.95) as well as from DDLPS cell lines. This MDM2 DNA could be transferred to preadipocytes (P-a), a major and ubiquitous cellular component of the DDLPS tumor microenvironment (TME), with subsequent P-a production of matrix metalloproteinase 2 (MMP2), a critical component in the metastatic cascade. From here the hypothesis that the DDLPS microenvironment (specifically P-a cells) may participate in DDLPS recurrence events. Since multifocal loco-regional DDLPS spreading is the main cause of the remarkably high lethality of this disease, a better understanding of the underlying oncogenic processes and their regulatory mechanisms is essential to improve the outcome of this devastating disease.

## INTRODUCTION

**Figure 1:**
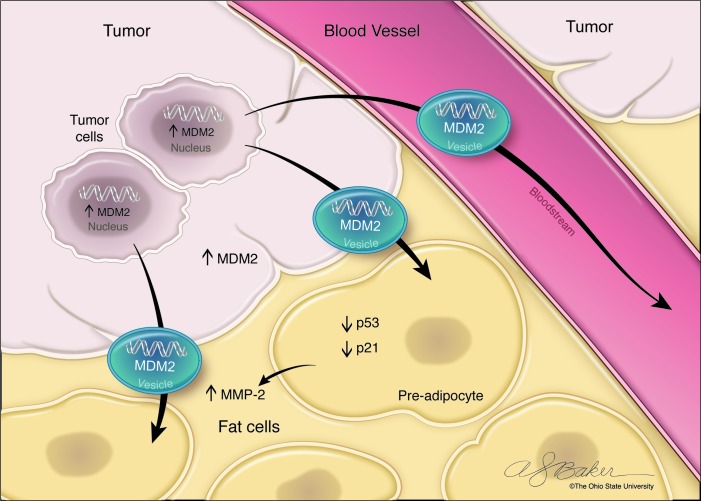
EV- dependent MDM2-DNA transfer from DDLPS to recipient P-a. We have demonstrated high levels of *MDM2* DNA in EVs derived from both DDLPS cell lines and also DDLPS patient serum samples. DDLPS EV *MDM2* can be transferred to recipient P-a where they promote release of active MMP2, thereby possibly contributing to subsequent multifocal loco-regional multifocal DDLPS dissemination and recurrence.

Dedifferentiated liposarcoma (DDLPS) of the retroperitoneum kills patients due to incontrollable loco-regional recurrence. In as many as 60% of patients such recurrences are multi-focal and are usually beyond meaningful therapeutic interventions other than palliation; hence an overall DDLPS survival rate of only 10-20% at 10 years [1-3]. The study of DDLPS molecular mechanisms has established the prevalence of 12q13-15 chromosomal amplification as the most common molecular derangement in this disease. As a result of this amplification, overexpression of CDK4, JUN and MDM2 occurs [4]. The latter is observed in almost 100% of DDLPS patients; MDM2 overexpression represses p53-induced transcription and also triggers ubiquitination and subsequent degradation of p53, the guardian of the genome. These processes contribute to tumor development in that enhanced MDM2 leads to impaired p53 suppression of tumor growth and impaired p53 promotion of apoptosis. While these molecular mechanisms underlying DDLPS pathogenesis has been previously described [5], the processes driving DDLPS recurrence, especially multi-focal recurrence, are yet to be determined. Given the potential therapeutic implications, we have been interested in possible mechanisms of DDLPS recurrence and are considering the role of the DDLPS microenvironment. The DDLPS microenvironment includes macrophages, adipocytes, and preadipocytes (P-a), all of which are likewise prevalent in the fat-bearing retroperitoneal compartment, the most common DDLPS location [6, 7]. We have discovered that DDLPS extrude extracellular vesicles (EVs), and they are involved in intercellular communication between DDLPS and recipient microenvironment cells such as P-a [8]. This interaction can result in transfer of protein, nucleic acids, and other biologically active molecules, and may contribute to tumor progression [8]. DDLPS EVs are transferred to recipient microenvironment P-a cells [8]. When exposed to DDLPS EVs, P-a can exhibit some phenotypic characteristics of cancer cells; e.g., enhanced proliferation and migration compared to cells incubated with EV-depleted medium. We discovered that *MDM2* DNA, amplified in nearly 100% of DDLPS, is transferred from DDLPS cells via EVs to P-a cells. P-a that are exposed to DDLPS EVs undergo a reduction of p53 and p21 levels (Figure 1). When P-a were incubated with EVs in the presence of the MDM2 inhibitor SAR405838 (Sanofi-Aventis), the rate of proliferation and migration of recipient cells was significantly impaired compared to P-a treated with EV alone. To date, transfer of material from EVs to recipient cells has been well documented for mRNAs, miRNAs, and proteins [9-12] but only rarely for DNA [13-15], especially regarding transfer of functional DNA [16]. We suggest the possibility that EVs can transfer functional DNA from host to recipient cells, perhaps serving in a manner analogous to a viral vector. If this hypothesis is correct, it would suggest many new questions about the mechanisms involved in such transfer, translation, and consequent transduction of protein from donor cell EVs to host cells. Although previously not considered, the DDLPS microenvironment (specifically P-a cells) may participate in DDLPS recurrence events. Extracellular matrix degradation is one of the key initial events underlying tumor cell dissemination and recurrence [17]. Responsible factors include metalloproteinases such as MMP2; these have been shown to promote cancer progression due to their degrading basement membrane components and collagen break down into peptides that act as chemoattractants for circulating tumor cells [18]. In the context of liposarcoma, MMP2 has been correlated with cell invasiveness, metastasis, and grade [19, 20]. We found that *MDM2* DNA transfer from DDLPS to P-a induces activation of P-a MMP2 (Figure 1). In keeping with our hypothesis, given the prevalence of P-a in the DDLPS microenvironment and throughout the retroperitoneum, stimulated P-a MMP2 activity may be pertinent to the extremely high rate of DDLPS multifocal recurrence. The need for prognostic biomarkers in a disease for which there are none is also apparent. Amplification of *MDM2* DNA is readily demonstrable by fluorescence *in situ* hybridization (FISH) and it commonly used to diagnose DDLPS [21]. We have shown that circulating EV-MDM2 DNA copy number can effectively discriminate DDLPS patients versus normal controls by Receiving Operating Characteristic curve analysis [8]. In these studies, the area under the curve (AUC) for *MDM2* was 95.8% with a 95% confidence interval, indicating robust separation of DDLPS patients from healthy controls. We are now conducting a clinical trial to more firmly establish EV *MDM2* DNA copy number as a useful DDLPS biomarker. The underlying regulatory mechanisms of this novel MDM2:MMP2 interactions are now the focus of our ongoing studies. We are considering whether the regulation of MMP2 by MDM2 might happen at the DNA, mRNA and/or protein level, and whether this regulation might occur in either a direct or perhaps indirect fashion involving other intermediate proteins such as TIMPs. In addition, the possible contribution of others MMPs (such as MMP9) to these processes is also currently being explored. Cracking the riddle of this rare and devastating disease is both a challenge and an imperative; please join us!
